# Relationships between inflammatory cytokine and cortisol responses in firefighters exposed to simulated wildfire suppression work and sleep restriction

**DOI:** 10.14814/phy2.12604

**Published:** 2015-11-24

**Authors:** Alexander Wolkow, Brad Aisbett, John Reynolds, Sally A Ferguson, Luana C Main

**Affiliations:** 1 Centre for Physical Activity and Nutrition Research, Deakin UniversityBurwood, Victoria, Australia; 2 Bushfire Co-Operative Research CentreEast Melbourne, Victoria, Australia; 3 Biostatistics Unit, Faculty of Health, Deakin UniversityBurwood, Victoria, Australia; 4 Central Queensland University, Appleton InstituteWayville, South Australia, Australia

**Keywords:** Cortisol, cytokines, firefighters, physical work, sleep restriction

## Abstract

The interplay between inflammatory and cortisol responses modulates an appropriate response to a stressor. Exposure to severe stressors, however, may alter the actions and relationships of these responses and contribute to negative health outcomes. Physical work and sleep restriction are two stressors faced by wildland firefighters, yet their influence on the relationship between inflammatory and cortisol responses is unknown. The aim of the present study was to quantify the relationship between the cytokine and cortisol responses to sleep restriction while performing simulated physical wildfire suppression work. Firefighters completed 3 days of simulated physical firefighting work separated by either an 8-h (Control condition; *n* = 18) or 4-h sleep (Sleep restriction condition; *n* = 17) opportunity on each of the two nights. Salivary cortisol and inflammatory cytokines (IL-6, IL-8, IL-1*β*, TNF-*α*, IL-4, and IL-10) were measured throughout each day. An increase in morning IL-6 was related to a rise (6.2%, *P *=* *0.043) in evening cortisol among firefighters in the sleep restriction condition. Higher morning IL-6 levels were related to increased (5.3%, *P *=* *0.048) daily cortisol levels, but this relationship was not different between conditions. Less pronounced relationships were demonstrated between TNF-*α*, IL-10, IL-4, and cortisol independent of the sleep opportunity, but relationships did not persist after adjusting for demographic factors and other cytokines. These findings quantify the relationship between cytokine and cortisol responses among wildland firefighters exposed to simulated occupational stressors. Potential disturbances to the IL-6 and cortisol relationship among sleep-restricted firefighters’ supports further investigations into the negative health effects related to possible imbalances between these systems.

## Introduction

A fundamental relationship exists between the immune and endocrine systems to modulate an adequate response to physiological and psychological stressors (Mcewen et al. 1997; Turnbull and Rivier 1999; Elenkov and Chrousos 2002; Elenkov 2008). The precise physiological mechanisms underlying this relationship are not fully understood. Evidence does suggest that activation of a bidirectional feedback loop between the end-products, cytokines and cortisol, is central to the appropriate functioning of the hypothalamic–pituitary–adrenal (HPA) axis, while maintaining homeostasis of the immune system (Turnbull and Rivier 1999; Petrovsky 2001). For example, exposure to a stressor and the subsequent release of certain inflammatory cytokines such as tumor necrosis factor-alpha (TNF-*α*), interleukin (IL)-10, and IL-6 activate the HPA axis and cause the release of cortisol (Turnbull and Rivier 1999; Steensberg et al. 2003). The anti-inflammatory effects of cortisol then feedback and suppress further release of cytokines (Riechlin 1993; Chrousos 1995; Elenkov and Chrousos 1999; Turnbull and Rivier 1999).

While the effect of cortisol on the immune system has been typically shown to be immunosuppressive in nature, cortisol can also be immune modulatory (Mcewen et al. 1997; Elenkov and Chrousos 2002; Elenkov 2008; Desantis et al. 2012). For instance, exposure to a chronic or severe stressor can cause prolonged activation of the HPA axis and excessive cortisol release, which is thought to contribute to inflammation by impairing the function of glucocorticoid receptors (e.g., downregulation, reduced expression, nuclear translocation; Mackin and Young 2004; Kunz-Ebrecht et al. 2004; Silverman and Sternberg 2012). Glucocorticoid receptor abnormalities reduce the immune system’s capacity to respond to cortisol and lower inflammation, resulting in concurrently sustained levels of cytokine and cortisol release (Chrousos 1995; Elenkov and Chrousos 1999; Miller et al. 2002). Altered immune–endocrine interactions have been linked to adverse health outcomes including coronary artery disease (Nijm and Jonasson 2009) and depression (Haddad et al. 2002; Lutgendorf et al. 2008). It is therefore important to further explore cytokine and cortisol relationships to provide evidence-based recommendations for firefighting populations where there is a high prevalence of mood disorders (Carey et al. 2011; Cook and Mitchell 2013; An et al. 2015) and cardiovascular disease (CVD)-related events and/or risk factors (Kales et al. 2007; Wolkow et al. 2014).

Research has demonstrated that the combined stressors of sleep restriction and physical work, which are common in emergency service occupations such as firefighting (Aisbett et al. 2012), can result in either increased cortisol (Opstad and Aakvaag 1981; Opstad 1994; Wolkow et al. 2015a) or altered cytokine levels (Gundersen et al. 2006; Lundeland et al. 2012; Abedelmalek et al. 2013; Wolkow et al. 2015b). However the degree to which cytokine and cortisol activities are related in the context of emergency work demands is unknown. Research has focused on a large population of healthy adults (Desantis et al. 2012), clinical samples, and/or experimental manipulation, such as exogenous cortisol or acute exercise (Derijk et al. 1997; Miller et al. 2002; Nijm and Jonasson 2009; Pledge et al. 2011). Findings in response to physical stressors have been equivocal. For instance, following a graded treadmill exercise test to 100% VO_2_max, cortisol appeared to suppress IL-1*β* and TNF-*α*, but had no effect on IL-6 (Derijk et al. 1997). In contrast, a more recent study by Pledge et al. (2011) found no association between cortisol and IL-6 in response to a 45-min resistance exercise protocol performed in the morning and evening. Furthermore, a review by Gómez-González et al. (2012) proposed that because sleep loss alters hormone and cytokine release, sleep loss further compromises the integrity of immune–endocrine interactions, though research examining the relationships between responses is needed. Firefighters exposed to multiple days of physical firefighting work separated by either an 8-h or restricted 4-h sleep exhibited significant changes in inflammatory cytokines (IL-6, TNF-*α*, IL-8, IL-1*β*, and IL-4) and daily cortisol levels (Wolkow et al. 2015a,b). Determining if these changes in cortisol and cytokine levels are related is important in understanding the physiological interactions underlying these responses to physical work and sleep restriction. Further knowledge of immune–endocrine relationships may help identify early indicators of chronic health outcomes associated with a dysregulation between inflammatory and cortisol responses (Haddad et al. 2002; Lutgendorf et al. 2008; Nijm and Jonasson 2009).

Therefore, the aim of the present study was to examine whether there was a relationship between cytokines and morning, evening, and total daily cortisol output, and if the observed relationships were altered by sleep restriction. To quantify potential relationships between physiological responses, cytokine and cortisol samples were obtained simultaneously at frequent intervals among firefighters completing a 3-day and 2-night simulated fire-ground deployment, with and without sleep restriction each night. Specifically, we hypothesized that sleep-restricted firefighters would have increased IL-6, IL-1*β*, IL-8, and TNF-*α* related to higher cortisol levels.

## Materials and Methods

### Participants

This study was based on data from 30 male and five female wildland firefighters (32 volunteer, three salaried personnel) who underwent a simulated 3-day fire-ground deployment. Participants were recruited from Australian fire agencies and included in the study if they had not been diagnosed with any form of respiratory or sleep disorders, heart disease, or diabetes. For purposes of analyses, participants were matched for age, sex, and body mass index (BMI) and then randomly assigned to either a control (CON) or sleep restriction (SR) condition. There were no significant differences between groups in BMI (*P *=* *0.113), age (*P *=* *0.913), presimulation physical activity levels (*P *=* *0.372), or years of firefighting experience (*P *=* *0.593; Table1). Participants completed a short occupational and firefighting history questionnaire and were assessed pre- and posttesting to exclude anyone who sustained an injury or became ill directly prior or during testing that could influence the inflammatory or cortisol levels measured and confound any subsequent comparisons. One firefighter in the SR condition withdrew due to injury and therefore, a final sample of 17 firefighters in the SR condition and 18 firefighters in the CON condition completed this study. Participation was voluntary and all firefighters provided written informed consent prior to commencing data collection. This study was approved by the institutions Human Research Ethics Committee and all procedures were in accordance with the Helsinki Declaration of 1975, as revised in 2008.

**Table 1:** Characteristics of firefighters in control (CON) and sleep restriction (SR) conditions

Characteristic	CON (*n* = 18)	SR (*n* = 17)
Age (years)	39 ± 16	39 ± 15
Men:Women	15:3	15:2
Weight (kg)	84.9 ± 17.8	93.8 ± 20.2
Height (cm)	178.1 ± 7.7	177.8 ± 7.4
BMI (kg/m^2^)	26.8 ± 5.0	29.6 ± 5.5
Firefighting experience (years)	6 (min–max 1.0–39.0)	10 (min–max 1.0–20.0)
Presimulation (16 h) physical activity (total activity counts)	305,233 ± 34,369	256,726 ± 19,609

BMI, body mass index; age, weight, height, and BMI are presented as mean ± standard deviation; firefighting experience is presented as median years and minimum–maximum years; For ease of interpretation, presimulation log physical activity data were back-transformed to total activity counts.

### Protocol and procedures

On arrival at the testing venue participants completed a familiarization session of the physical work tasks and physiological tests, followed by an adaptation night (8-h sleep opportunity) in the testing environment. Participants then completed a 3-day and 2-night simulated fire-ground deployment. On each night, participants in the CON condition had an 8-h sleep opportunity (i.e., bedtime 22:00–06:00), while participants in the SR condition had a 4-h sleep opportunity (i.e., bedtime 02:00–06:00). Sedentary factors such as extended travel time between the fire line and camp, difficulty sleeping in an unfamiliar and noisy environment at the camp site, and winding down after a shift can contribute to sleep restriction on the fire-ground (Cater et al. 2007). To reflect these conditions, participants in the SR condition were free to perform sedentary leisure activities (e.g., watching television, reading etc.) until the delayed bedtime. The duration of sleep restriction in this study was based on Australian wildland firefighters’ self-reported average sleep per rest period on the fire-ground (Cater et al. 2007). Both conditions received an 8-h recovery sleep opportunity after testing to ensure that all participants were rested before leaving the testing venue. Participants slept on camp beds in the simulated testing environment to replicate sleeping conditions when deployed to the fire-ground (Cater et al. 2007).

All testing procedures were performed in a 9 × 13 meter room that was climate-controlled. Windows were blacked out and ceiling room lights turned on during each wake period (i.e., lights on between 06:00 and 22:00 in the CON condition and 06:00 and 02:00 in the SR condition) and turned off during the sleep period (i.e., lights off between 22:00 and 06:00 in the CON condition and 02:00 and 06:00 in the SR condition). Therefore, participants were not time isolated, as they knew when it was night time and daytime, thus preventing the confounding influence a possible desynchronization between the external environment and internal physiological rhythms has on cortisol and cytokine levels. Throughout the protocol, the testing environment was maintained at moderate temperatures (18–20°C) in both conditions using split cycle air conditioners (Panasonic, Osaka, Japan). Ambient air temperature was monitored using a wireless temperature and humidity logger (HOBO ZW_003, Onset Computer Corporation, Bourne, MA), data receiver (HOBO ZW_RCVR, Onset Computer Corporation), and software (HOBO Pro Software, Onset Computer Corporation). Adhering to fire-ground practices, food and drink intake during the study was ad libitum and the amount of fluid ingested was recorded. These data were then extracted using the FoodWorks 7 nutrition software (2012 Xyris Software Pty Ltd, Kenmore Hills, Australia). Although food intake was recorded, the measurement of daily fluid consumption from food and drink combined has only been reported in the Results section.

**Figure 1:**
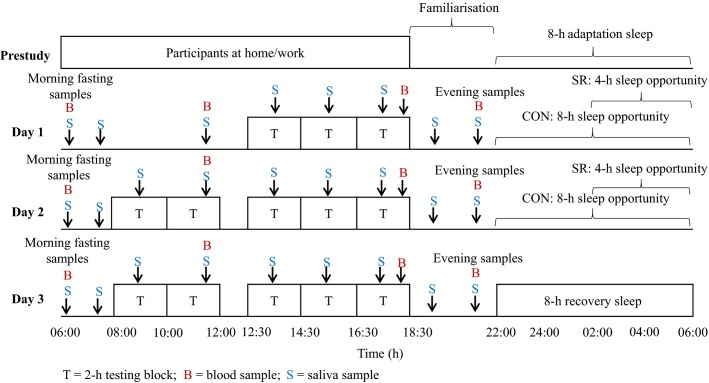
Study protocol for the control and sleep restriction condition.

Participants in both conditions were tested in groups of 3 to 5. All participants completed a 2-h testing block, three times on day 1 and five times on days 2 and 3 (Fig.1). Each testing block consisted of a 55-min work circuit simulating physical wildland firefighting work (Phillips et al. 2012), followed by physiological (20–25 min) and cognitive (20–25 min; reported in Christoforou et al. 2013) data collection periods and a 15- to 20-min rest period. The time allocated to physical work and rest periods described above reflects the physical activity profile observed across a shift on the fire-ground (Aisbett et al. 2007; Phillips et al. 2011; Raines et al. 2013).

#### Simulated physical firefighting work circuit

The physical work circuit comprised six simulated wildland firefighting tasks designed to mimic the physical demands involved in Australian wildfire suppression work (Ferguson et al. 2011; Phillips et al. 2012; Vincent et al. 2015). A job task analysis was used to design the firefighting work circuit. Each of the tasks identified for inclusion in the circuit have been verified by panels of firefighter subject matter experts (including senior operational firefighters and training officers) as being representative of the movements that encompass key firefighting tasks frequently performed on the fire-ground (Ferguson et al. 2011; Phillips et al. 2012). The tasks included lateral repositioning of a hose, rake hoe work, hose rolling, charged hose advance, black out hose work, and static hold of a hose. The performance of each physical task (i.e., repetitions completed for each task within each work period) was self-paced and completed in a predetermined order with work-to-rest ratios designed to mimic the performance of these tasks on the fire-ground (Ferguson et al. 2011; Phillips et al. 2015; Vincent et al. 2015).

#### Blood sampling and cytokine analysis

Fingertip capillary blood samples were collected to determine IL-6, IL-8, IL-1*β*, TNF-*α*, IL-4, and IL-10 cytokine levels in blood plasma at four time points each day: a fasting sample in the morning (i.e., 06:15), late morning (i.e., 11:30), evening (i.e., 18:15), and at night (i.e., 21:30; Fig.1). Prior to sample collection, participants held a heat pack in their hand to aid in blood flow to the fingertips. At each time point, a 500-*μ*L sample of whole blood was collected in to a microtainer coated with K_2_ EDTA (Becton Dickinson ref: 365974). Whole blood samples were centrifuged for 10 min at 5000 revolutions/min (83 Hz) and the plasma was separated and stored at <−80°C. Although previous emergency service-based studies have used venous blood samples when investigating cytokine levels (Bøyum et al. 1996; Gundersen et al. 2006; Lundeland et al. 2012), capillary blood samples were chosen because it is a minimally invasive method to conveniently obtain multiple daily blood samples from participants wearing personal protective clothing and performing repeated bouts of physical work. Some studies suggest that, due to a small local inflammatory response to the pinprick, capillary blood samples can result in higher cytokine levels (Eriksson et al. 2007; Cullen et al. 2015). However, recent evidence indicates a close correlation between venous and capillary plasma IL-6 responses at rest (Faulkner et al. 2014), as well as during and postexercise (Faulkner et al. 2014; Cullen et al. 2015). Conversely, other reports have found that venous and capillary concentrations of TNF-*α* (Eriksson et al. 2007) and IL-6 (Cullen et al. 2015) differed at rest. However, these studies (Eriksson et al. 2007; Cullen et al. 2015) did not control factors known to impact resting cytokine levels such as the time of day the sample was taken or whether or not the sample was taken under fasting conditions (Zhou et al. 2010). Control over these factors in the current study limits their potentially confounding influence on cytokine levels at rest.

The Milliplex Human MAP Cytokine immunoassay kit (Millipore, Billerica, MD) was used to profile the expression of inflammatory markers in the plasma samples. The assay was performed according to the manufacturer’s instructions on the Bioplex 200 array reader (V.5.0, Bio-Rad Laboratories, Hercules, CA). This involved ensuring that quality control samples were run with each cytokine assay. The minimal detectable concentrations were 0.06, 0.42, 0.20, 0.05, 0.48, and 0.07 pg/mL for IL-1*β*, IL-4, IL-6, IL-8, IL-10, and TNF-*α*, respectively. Cytokines intra- and interassay coefficients of variation (CVs) were within acceptable ranges (intra-assay: 4.5–10.0%; interassay: 9.8–20.5%) for all analytes (CV <25%; Findlay et al. 2000; Chowdhury et al. 2009) and comparable to CVs reported for cytokines sampled using venous blood in previous exercise-based literature (Abedelmalek et al. 2013; Faulkner et al. 2014; Cullen et al. 2015).

#### Saliva sampling and cortisol analysis

Salivary samples were collected using a cotton swab (Salivette; Sarstedt, Nümbrecht, Germany) at baseline (i.e., 07:30) and at the completion of each physical work circuit (i.e., 09:00, 11:15, 13:30, 15:30, 17:30). Further daily samples were taken in both conditions after awakening (i.e., 06:30) and in the evening (i.e., 19:30, 21:30; Fig.1). To prevent sample contamination, participants were not allowed to eat or drink 15 min prior to saliva collection. All samples were stored at <−80°C. Samples were then thawed and centrifuged for 10 min at 5000 revolution/min (83 Hz) before assessing salivary cortisol concentration using a high-sensitivity enzyme immunoassay ELISA kit (IBL International, Hamburg, Germany). The assay was performed according to the manufacturer’s directions and read at 450 nm on a luminescence microplate reader (Synergy™ 2 SL, BioTek, Winooski, VT). Analytical sensitivity (lower limit of detection) was 0.14 nmol/L and the intra- and interassay CVs were 7.2% and 10.7% (both mean 13.8 nmol/L), respectively, which are within the acceptable ranges (i.e., accuracy <15%; intra-assay CV <10%; interassay CV <15%; Biopharmaceutics Coordinating Committee, 2001, Nicolson 2008).

#### Sleep and physical activity monitoring

Participants’ sleep was recorded using the Siesta Portable EEG system (Compumedics E-Series; Melbourne, Victoria, Australia) and standard polysomnographic (PSG) montage. Each night, PSG recording began at 21:00 for both conditions. From each sleep period, participants’ total sleep time (minutes) was calculated. In addition, participants wore activity monitors (*Actical* MiniMitter/Respironics, Bend, OR) to measure sleep across the two nights prior to the study. Further information on participants’ physical activity 16 h prior to the simulation was provided through the use of activity monitors, which were set to sample in 1-min epochs, with a sensitivity of <40 counts per epoch to distinguish between sleep and wake states (Darwent et al. 2008). Activity data were downloaded using Actical software (version 3.10, MiniMitter/Respironics, Bend, OR) and analyzed and expressed using total activity counts.

### Statistical analyses

Prior work from this sample has examined cortisol and cytokine responses separately (for cortisol findings, see Wolkow et al. 2015a; and for cytokine findings, see Wolkow et al. 2015b). This study focuses on the relationships between cytokine and cortisol activity from this same sample of firefighters. Prior to the analysis, cytokine values >2 standard deviations above (IL-4 = 5% of sample; IL-6 = 6% of sample; IL-1*β* = 6% of sample; IL-8 = 6% of sample; IL-10 = 5% of sample; TNF-*α *= 4% of sample) the mean were considered outliers and subsequently removed (Finnerty et al. 2008; Nguyen et al. 2010). Values below the detectable range of the Milliplex Human MAP Cytokine immunoassay kit (IL-4 = 18% of sample; IL-6 = 0.9% of sample; IL-1*β* = 0.2% of sample; IL-8 = 0.7% of sample; IL-10 = 1.4% of sample; TNF-*α *= 0.2% of sample) were replaced with the minimal detectable concentration as advised in the protocol (Weiskopf et al. 2009; Liberati et al. 2013). With the exception of TNF-*α* (for which raw values achieved normality and homogeneity of variance), all cytokine, cortisol, and activity count measurements were adjusted using a natural log transformation to achieve normality, assessed using the Shapiro–Wilk test (*P *>* *0.05). Normality and homogeneity of variance of the residuals were further assessed by inspection of the resulting mixed model analysis (Field 2009). The resultant diagnostic plots revealed no departures from these required assumptions. Due to the sampling design, a measurement for cortisol at 09:00 was missing for all participants on day 1. Consequently, missing value codes were appended to the data set, but this did not affect the statistical analyses of the data.

Sleep duration, physical activity, and demographic characteristics were analyzed with the analysis of variance (ANOVA) method using GenStat software (GenStat *for Windows* 16.1 Edition; VSN International, Hemel Hempstead, UK). To assess the possible relationships between inflammatory and cortisol responses, cytokine levels were analyzed in relation to cortisol parameters which included morning (i.e., 06:30) and evening (i.e., 21:30) cortisol levels and total daily cortisol output. Total daily cortisol was determined using the area under the curve (AUC) with respect to ground, which was calculated for each participant on each day using the trapezoidal method (Pruessner et al. 2003). To investigate possible relationships, the cortisol parameters were modeled as a function of morning fasting cytokine levels (i.e., 06:15) and across daily cytokine levels (i.e., 06:15, 11:30, 18:15 and 21:30) in separate models. For repeated cortisol and cytokine measurements nested within days, subjects, and groups, linear mixed models (LMM) were fitted by the restricted maximum likelihood (REML) method (Payne et al. 2011) using GenStat software (GenStat *for Windows* 16.1 Edition; VSN International, Hemel Hempstead, UK). This method allows for the possibility of autocorrelation in the repeated cytokine or cortisol measurements (i.e., days) on each individual by including a model for the covariance structure.

The final “full” model fitted to morning, evening, and AUC cortisol parameters included potential fixed and interaction effects critical to the design of the study, which included condition and day, along with a two-way interaction of condition by day. To investigate possible relationships between the cytokine measures and cortisol parameters, this final model fitted potential fixed effects of each cytokine along with two-way interactions of condition by cytokine, day by cytokine, and potential three-way interactions of condition by day by cytokine. For each model, random effects of group, profile (or participant) and a group by profile interaction were investigated both without (i.e., Independence model) and with an unstructured covariance model for the within-subject autocorrelation.

Models fitted to the cortisol parameters investigated each of the cytokines separately and all in one model to examine their independent relationships with each of the cortisol variables. All models were fit with and without controlling for the demographic factors of sex, age, and BMI. Model fit was assessed by Akaike’s information criterion (AIC) and small differences (∆AIC) in this criterion compared to other candidate models were used to identify parsimonious models (Burnham and Anderson 2002). Statistical significance was set at *P *<* *0.05 and slopes of potential interactions are represented using regression (unstandardized) coefficients (*b*) and mean percentage changes presented.

## Results

### Sleep, prestudy physical activity, and daily fluid consumption

Sleep duration in the two nights prior to the study was not significantly different to the adaptation night or between conditions (*P *>* *0.05; Table2). The average total sleep time measured for both conditions was similar on the adaptation night (*P *>* *0.05; Table2). During nights 2 and 3 of the simulation, total sleep time was as expected, given the sleep opportunity provided in the CON and SR conditions (*P *<* *0.001; Table2). Furthermore, there were no differences between conditions in participants’ physical activity levels in the 16-h prior to beginning the study (total physical activity counts *P *>* *0.05; Table1). The firefighting history questionnaire also revealed that no firefighters attended a firefighting emergency in the 24 h prior to beginning the study. No between-condition differences in prestudy sleep and physical activity/work ensures that on entering the study, both groups had experienced a similar level of acute stressors, minimizing the impact that prior exposure could have on subsequent cortisol and cytokine measures. In addition, participants’ mean daily fluid intake was similar between conditions on each of the testing days (*P *>* *0.05).

**Table 2:** Total sleep time (mean ± SD) for each night in both conditions (h)

Night	CON	SR
Prestudy 1	7.3 ± 1.4	6.7 ± 0.9
Prestudy 2	6.7 ± 1.3	6.2 ± 1.4
1 (adaptation)	6.3 ± 0.9	6.4 ± 0.7
2	6.9 ± 0.4	3.6 ± 0.2*
3	6.9 ± 0.5	3.7 ± 0.2*

* *P *<* *0.001 between conditions. CON, control; SR, sleep restriction.

### Cortisol and cytokine relationships

The LMM model with the lowest AIC for each of the cytokines was the full fixed effects independence model which, after inspection of the ∆AIC, had the best fit to model the relationship between cytokine and cortisol parameters. While there were no significant relationships between any of the cytokines investigated and morning cortisol levels (*P *>* *0.05), significant relationships between morning cytokine levels and cortisol AUC were demonstrated. A main effect for IL-6 indicated that higher morning levels of this cytokine were positively related with greater cortisol AUC levels independent of condition (*P *<* *0.001; Table3). For this relationship, a 1 standard error unit increase in morning IL-6 was related to a 5.3% rise in AUC and a 5.5% increase after controlling for demographic factors (*P *=* *0.04; Table3). The relationship between morning IL-6 and cortisol AUC remained significant after controlling for the other cytokines and demographic factors (*P *=* *0.048; *b* = 0.4923, SE = 5.921), demonstrating that the association is independent of IL-8, IL-1*β*, TNF-*α*, IL-4, and IL-10, and BMI, sex, and age. A further two-way interaction of condition by morning IL-6 for cortisol AUC indicated a steeper rise in AUC for the SR condition when IL-6 levels increased (*P *=* *0.033; SR *b* = 4.324, SE = 0.816; CON *b* = 1.889, SE = 1.122). However, this interaction was no longer significant after controlling for demographic factors (*P *>* *0.05).

**Table 3:** Main effects of each cytokine with cortisol AUC with and without controlling for demographic factors

Parameter		Morning fasting sample					
		IL-6	TNF-*α*	IL-8	IL-1*β*	IL-10	IL-4
Models uncontrolled for demographic factors (i.e., age, BMI, and sex)							
Cortisol AUC	*b* (SE)	1.525 (1.104)	0.386 (0.238)	0.149 (1.296)	0.207 (0.700)	−0.676 (1.412)	−0.571 (0.589)
	*F*	15.42	4.01	0.11	0.27	4.94	0.80
	*P*	<0.001	0.049	0.738	0.602	0.029	0.372
	%	5.3	0.3	0.6	0.4	−2.7	−1.2
Models controlled for demographic factors (i.e., age, BMI, and sex)							
Cortisol AUC	*b* (SE)	1.337 (1.244)	0.270 (0.291)	0.905 (1.295)	1.275 (0.856)	−3.539 (1.575)	−0.847 (0.666)
	*F*	4.41	1.00	0.49	2.76	0.44	0.06
	*P*	0.040	0.321	0.486	0.103	0.509	0.801
	%	5.5	0.3	4.3	4.4	−14.3	−2.3

AUC, area under the curve; BMI, body mass index; *b*, regression (unstandardized) coefficients; SE, standard error.

Significant relationships between the morning TNF-*α* and IL-10 with cortisol AUC were also demonstrated. For instance, positive (increasing) cortisol AUC levels were related to a rise in morning TNF-*α* (*P *=* *0.049; 0.3% increase in AUC; Table3). Conversely, there was a negative (decreasing) association for cortisol AUC when morning IL-10 levels increased (*P *=* *0.029; 2.7% decrease in AUC; Table3). But when controlling for other cytokines and demographic factors, the relationships for morning IL-10 and TNF-*α* were no longer significant (*P *>* *0.05). No significant interactions involving condition and/or day were found when investigating the relationship between these cytokines and cortisol AUC, nor were there relationships between morning levels of IL-4, IL-1*β*, and IL-8 with cortisol AUC.

Investigation of possible relationships between cytokine profiles measured across the day (i.e., 06:15, 11:30, 18:15, and 21:30) and cortisol AUC revealed significant relationships involving the IL-6 and IL-10 daily profiles, independent of the sleep opportunity. A main effect indicated positive (increasing) cortisol AUC levels when IL-6 increased across the day (*P *=* *0.047; *b* = 0.240), but this interaction did not persist after controlling for demographic factors and other inflammatory cytokines. When demographic factors were adjusted for a significant interaction between day and daily IL-10 measurements (*P *=* *0.023) indicated that higher levels of IL-10 were related to an increase in cortisol AUC on day 2 (*b* = 0.892), while slight negative and positive relationships were demonstrated for this parameter on days 1 (*b* = −0.103) and 3 (*b* = 0.152), respectively. But this relationship did not persist after controlling for other cytokines (*P *=* *0.07).

Interactions between condition and morning IL-6 levels were demonstrated for evening cortisol. In the SR condition, this interaction showed that a rise in morning IL-6 was related to an elevation in cortisol that evening, after controlling for demographic characteristics (*P *=* *0.043; *b* = 0.300, SE = 0.154). In the CON condition, however, this interaction indicated that when morning IL-6 levels increased, evening cortisol levels decreased slightly (*b* = −0.136, SE = 0.210). A 1 standard error unit increase in morning IL-6 resulted in a 6.2% rise in evening cortisol for the SR condition and 2.5% decrease in this parameter for the CON condition. The relationships were significant but partially attenuated after controlling for the other cytokines (5.7% increase in SR, *b* = 0.876, SE = 0.288; 0.5% decrease in CON, *b* = −0.914, SE = 0.332; *P *=* *0.039). A further two-way interaction indicated that a rise in IL-4 levels was related to positive (increasing) evening cortisol levels in the CON condition (*b* = 0.110), while in the SR condition this interaction demonstrated negative (decreasing) evening cortisol levels when IL-4 increased (*b* = −0.081; *P *=* *0.045). However, this relationship was no longer significant after adjustment for demographic factors (*P *>* *0.05).

## Discussion

Findings from the current study quantify the relationship between cytokine and cortisol levels among firefighters exposed to simulated occupational demands. In response to the combined stressors of shortened sleep and physical work, an increase in morning IL-6 was related to a rise in evening cortisol among firefighters in the SR condition and decreased evening cortisol in the CON condition. This relationship remained significant when controlling for other cytokines and demographic factors. A positive association was also demonstrated in the SR condition between the daily IL-6 profile and cortisol AUC, but this did not persist when demographic factors were included in the analyses. After controlling for demographic factors, a rise in morning IL-6 was found to further relate to increased cortisol AUC, independent of condition. Less pronounced relationships were also demonstrated between TNF-*α*, IL-10, IL-4, and cortisol.

When the firefighters were sleep restricted, the detected relationship between IL-6 and cortisol may be reflective of how elevated IL-6 stimulates increased corticotropin-releasing hormone secretion as well as arginine vasopressin and other corticotropin secretagogues (Chrousos 1995; Petrovsky 2001), which lead to the more pronounced increase in evening cortisol. However, an 8-h sleep opportunity between shifts alters this relationship so that a rise in IL-6 no longer relates to increased evening cortisol. It is possible the interactions revealed here between IL-6 and cortisol may explain how restricted sleep and physical work in our previous study resulted in an elevated cortisol profile in the afternoon and evening when compared to physical work and an 8-h sleep (Wolkow et al. 2015a). Elevated evening (and afternoon) cortisol has been linked to insulin resistance and impaired memory (Dallman et al. 1993; Mcewen 1998; Spiegel et al. 1999). The current study therefore suggests that despite a rise in morning IL-6, an 8-h sleep opportunity between firefighting shifts mitigates subsequent increases in evening cortisol, offering a protective buffer against adverse health effects.

A positive relationship, between IL-6 and evening cortisol in the sleep-restricted condition and cortisol AUC independent of condition, appears in contrast to the anti-inflammatory effects of this hormone (Chrousos 1995). However, this finding is consistent with research suggesting that IL-6 is more resistant to the effects of cortisol under physical stress (i.e., exercise test to 100% VO_2_max; Derijk et al. 1997). In contrast, no relationships were found between IL-6 and cortisol among healthy males following acute resistance training (Pledge et al. 2011). Meanwhile, to our knowledge, this is the first study to have investigated how sleep influences cytokine and cortisol relationships. The limited and equivocal findings highlight the need for further research conducted among larger samples of healthy adults that examines the relationship between cytokine and cortisol responses to physical work and sleep restriction. Findings in older adults and clinical populations indicate that disturbances to the normal actions of cortisol and cytokines result in elevated IL-6 and evening cortisol and cortisol AUC (Nijm et al. 2007; Lutgendorf et al. 2008; Nijm and Jonasson 2009), similar to that demonstrated among the current group of firefighters.

Excessive long-term exposure to cortisol may downregulate hormonal receptors and thereby impair the immune systems response to cortisol’s anti-inflammatory actions (Chrousos 1995; Elenkov and Chrousos 1999; Miller et al. 2002; Desantis et al. 2012). Accordingly, repeated firefighting deployments across a fire season which expose personnel to sleep restriction and physical work could prolong the observed alterations in IL-6 and evening cortisol, resulting in negative physical (e.g., CVD; Nijm et al. 2007) and mental health outcomes (e.g., depression; Lutgendorf et al. 2008; Haddad et al. 2002). Therefore, acute immune–endocrine relationships observed in the current study could be an early indicator of chronic, firefighter-relevant health outcomes associated with dysregulation to these systems. For instance, high levels of depression have been reported among firefighters (Carey et al. 2011; Cook and Mitchell 2013; An et al. 2015), while fire suppression activities were associated with an increased risk of death from coronary heart disease (Kales et al. 2007).

Short-term elevations in HPA axis and immune activity have also been related to acute changes in mood (Kemeny 2007; Vgontzas et al. 2008). Mood has been shown to impact factors important to occupational settings such as worker helpfulness (Carlson et al. 1988), job satisfaction (Fisher 2000), and the probability of making an error (Appel et al. 1980). For example, Christoforou et al. (2013) found that compared to firefighters who had an 8-h sleep, those who had a 4-h sleep opportunity demonstrated reduced attention over the multiday firefighting simulation. Therefore, it is important to determine how, in response to sleep restriction and physical work, acute changes in mood may relate to disturbances between cytokine and cortisol interactions in the short-term. However, based on the bidirectional relationship between cytokines and cortisol (Turnbull and Rivier 1999; Petrovsky 2001), further research including experimental alterations to cortisol or cytokine levels is also needed to understand the causal direction of the observed relationships.

The positive association between morning IL-6 and cortisol AUC (5.5% increase) independent of sleep is consistent with Desantis et al. (2012) who reported a similar increase (6.5%) in AUC with elevated levels of IL-6 among healthy adults examined under naturalistic settings. Adjustment for levels of other cytokines and demographic factors preserved the positive association between IL-6 and cortisol AUC for firefighters. Similar increases in cortisol AUC have been associated with elevated levels of daily negative affect (i.e., 11%; Piazza et al. 2013), but it is currently unclear how magnitude changes in AUC relate to other health outcomes (e.g., atherosclerosis, metabolic syndrome). The detected rise in firefighters’ cortisol AUC after controlling for covariates was slightly less than that reported (7.4% in AUC) by Desantis et al. (2012) who controlled for TNF-*α* and IL-10. Furthermore, Desantis et al. (2012) controlled for additional confounders (e.g., race/ethnicity, income/wealth, physical activity level) that were not measured among the current population of firefighters. Failure to record and control for these behavioral and sociodemographic factors reported to influence the HPA axis (Ranjit et al. 2009; Hajat et al. 2010) is a limitation of the current study that could also explain the different rise in firefighters’ cortisol AUC.

Altered hydration levels lead to variations in plasma volume, which can further contribute to changes in cytokines and cortisol (Steptoe et al. 2007; Hill et al. 2008). Unfortunately plasma volume was not measured among firefighters and therefore unaccounted for in the current study. Despite this limitation, there were no differences in daily fluid consumption between conditions and fluid intake was similar to levels demonstrated among firefighters completing multiple days of physical work in the heat, reported to be hydrated as determined using urine specific gravity (Larsen et al. 2015). Moreover, daily water intake exceeds recommended levels for adult males performing exercise in mild conditions (Gunga et al. 1993; Kenefick and Sawka 2007). It is therefore unlikely that perturbations in hydration in the current study impacted cytokine and/or cortisol levels. Further understanding the relationships between these physiological responses among firefighters or other occupational groups exposed to physical work and sleep restriction requires additional hydration measures (i.e., plasma volume) and larger samples with sufficient power to examine a range of sleep durations and other potential sociodemographic and behavioral factors.

In the present study, relationships between TNF-*α*, IL-10, IL-4, and cortisol were less pronounced than for IL-6 and no longer significant after adjusting for demographic factors and/or other cytokines. Despite this, these findings are the first to show how occupational stressors influence the relationship between TNF-*α*, IL-10, IL-4, and cortisol, and therefore, should still be considered, but with caution. For instance, higher morning levels of TNF-*α* were related to an increase in cortisol AUC independent of the sleep opportunity across the simulation. While cortisol has been found to interact with TNF-*α*, findings are mixed with reports indicating that exogenous cortisol can potentiate TNF-*α* levels in rats (Frank et al. 2010) or suppress TNF-*α* among humans (Derijk et al. 1997). Stressor-induced cortisol levels, attained through maximal exercise (i.e., 100% VO_2_max), can also suppress TNF-*α* production (Derijk et al. 1997). Furthermore, across the simulation an increase in morning IL-10 was related to a decrease in cortisol AUC independent of the sleep opportunity. Higher daily levels of IL-10 were also related to an increase in cortisol AUC on day 2 of the simulation, while smaller negative and positive relationships were demonstrated for this parameter on days 1 and 3, respectively. Between-day differences provide further evidence for the role IL-10 plays in up- and downregulating the cortisol response to acute stressors (Elenkov and Chrousos 1999; Smith et al. 1999). Furthermore, the observed associations between, IL-10, TNF-*α*, and cortisol were independent of the sleep opportunity, highlighting that exposure to the physical work demands was the major stressor influencing these relationships in the current study. Consistent with our results that higher IL-4 is related to increased and decreased evening cortisol in the CON and SR conditions, respectively, evidence indicates that IL-4 affects cortisol release (Woods and Judd 2008) by inducing the expression of enzymes involved in regulating this hormone (Thieringer et al. 2001). However, because the relationship between firefighters’ IL-4, TNF-*α*, IL-10 and cortisol AUC did not persist after adjusting for demographic factors and/or other cytokines, further research should include larger samples with the statistical power to include covariates such as race/ethnicity, smoking, and physical activity (Desantis et al. 2012) in to the analyses.

While further relationships may exist between IL-8 and IL-1*β*, and cortisol (Derijk et al. 1997; Corsini et al. 2014), research involving IL-8 and IL-1*β*, and to an extent IL-4, has been centered on acute immune responses to more intense experimental stressors such as maximal exercise and large doses of exogenous cortisol and cytokines (Derijk et al. 1997; Thieringer et al. 2001; Corsini et al. 2014). Moreover, research investigating relationships between IL-8, IL-1*β*, and cortisol has, to date, been conducted in animal models or in vitro experiments. Therefore, in response to the moderate stressors studied, immune–endocrine interactions between these cytokines (i.e., IL-8, IL-1*β*, and IL-4) and cortisol are unlikely.

The current study matched firefighters in the CON and SR conditions for age, BMI, and gender. However, because a crossover design was not utilized, there is a possibility that intragroup differences may explain the divergent relationships found for IL-6 and cortisol between conditions. For example, firefighters in the SR condition may have had less experience performing the type of physical work tested when compared to the CON condition, thus resulting in the positive immune–endocrine relationship observed for the sleep-restricted firefighters. However, employing mixed models for the analyses, factor in unique responses of participants to an intervention (Van Dongen et al. 2004), and therefore, should account for the effects of individual subject variability. By closely replicating many aspects of a fire-ground deployment, the simulated environment further permitted the quantification of physiological stress responses during periods of controlled physical work and sleep restriction. For instance, the physical work tasks chosen simulate preparatory or postfire clean-up work that comprises a large component of firefighting, but is performed in cooler conditions (Budd et al. 1997; Raines et al. 2012). For instance, consecutive days of wildfire suppression work in temperatures ranging between 15.8 and 26.4°C have been reported during large campaign wildfires in parts of south eastern Australia (Raines et al. 2012). Moreover, the sleep durations, bedding, and beds in the current study mimic observed fire-ground conditions (Cater et al. 2007; Ferguson et al. 2011). Ensuring this high level of ecological validity for certain phases of a wildfire deployment make the detected findings applicable to the physical work (i.e., blacking out, carrying and dragging hoses and rake hoe work) and sleep involved during wildland firefighting in mild temperatures. However, the artificial setting limits the extrapolation of findings to other fireground demands which were not replicated, such as exposure to high ambient and radiant heat and smoke (or its constituent elements; Aisbett et al. 2012). External heat sources while performing physical work may influence inflammatory markers and cortisol (Lieberman et al. 2005; Walsh and Whitham 2006; Hailes et al. 2011). Wood smoke exposure has also been associated with increases IL-6 and IL-8 (Swiston et al. 2008), and decreases in IL-10 and cortisol (Burgess et al. 2002; Al-Malki et al. 2008). Therefore, investigating possible relationships between cytokine and cortisol levels among sleep-restricted firefighters performing physical work in a hot and smoky environment represents a logical next step for firefighting-based research in this area.

## Conclusion

When firefighters had restricted sleep while performing physical work, an increase in morning IL-6 levels were positively related to a rise in evening cortisol. Conversely, a rise in IL-6 was associated with a decline in evening cortisol when firefighters had an 8-h sleep between shifts. Given how elevated evening cortisol can have adverse consequences to health (Dallman et al. 1993; Mcewen 1998; Spiegel et al. 1999), a rise in IL-6 in the morning, but decreased evening cortisol may reflect normal “nondamaging” immune–endocrine function. Therefore, relationships described here highlight to fire agencies the important role an 8-h sleep opportunity between shifts has in preventing elevated evening cortisol. In addition, evidence of altered immune–endocrine function among sleep-restricted firefighters and those receiving a normal sleep while performing physical work supports additional investigation into the short- (e.g., changes in mood; Vgontzas et al. 2008; Kemeny 2007) and long-term (e.g., coronary artery disease and depression; Nijm and Jonasson 2009; Nijm et al. 2007; Lutgendorf et al. 2008; Karlovic et al. 2012) health effects associated with imbalances between these systems. Using larger sample sizes, further research should also determine the direction of relationships between cytokines and cortisol and the potential impact other factors (e.g., sociodemographic and behavioral) have on these systems.
